# Unraveling endocannabinoid signaling disruption in a preclinical model of neurodevelopmental disorders

**DOI:** 10.1172/JCI196707

**Published:** 2025-09-02

**Authors:** Nephi Stella

**Affiliations:** Departments of Pharmacology and Psychiatry and Behavioral Sciences, The University of Washington, Seattle, Washington, USA.

## Abstract

The search for transformative medicines has continuously uncovered select diseases associated with the disruption of the endocannabinoid (eCB) signaling system in the brain and emphasized the therapeutic value of small molecules that rescue this signaling system. In this issue of *JCI*, Wang et al. report that genetic disruption of PPP2R1A function in mouse forebrain, a preclinical mouse model of neurodevelopmental disorders, resulted in pronounced impairment of eCB signaling. Notably, small-molecule inhibitors of eCB inactivation rescued both eCB signaling and cognitive dysfunction in this model, providing a solid foundation to move such transformative therapeutic approaches based on targeting eCB signaling toward human clinical trial testing.

## Sourcing therapeutic targets from the endocannabinoid system

Δ^9^-Tetrahydrocannabinol (THC) is the principal bioactive ingredient in the *Cannabis* plant, acting as a partial agonist at the endogenous cannabinoid receptor CB1R. Though the earliest therapeutic use of THC was likely for its analgesic properties (1), its therapeutic benefits have been explored for a range of CNS indications, including epilepsy, migraines, insomnia, multiple sclerosis, and Huntington’s disease (2). Its efficacy for these indications was systematically quite limited, and THC induces nontrivial neurological and systemic side effects, reducing its therapeutic value (3). Moreover, chronic use of high amounts of THC impacts brain development and function and is associated with increased incidence of mental health disorders, including cannabis use disorder and psychosis or schizophrenia (4).

Targeting the endocannabinoid (eCB) signaling system may be a way to bypass the therapeutic limitations of THC. The eCB signaling system plays a fundamental role in the control of synaptic transmission and plasticity as well as other physiological processes (5, 6). The two main eCBs, anandamide and 2-arachidonyl glycerol (2-AG), are signaling lipids that activate CB1R on neurons and astrocytes and CB2R on immune cells. 2-AG is abundantly present in the CNS, and its levels and activity at CB1R are tightly and differentially controlled by the enzymes MAGL and ABHD6 (7). Several preclinical studies have reported on the therapeutic value of MAGL inhibitors for the treatment of pain and inflammation, ischemic stroke, migraines, and anxiety-related disorders (8–10). Thus, targeting eCB inactivation represents a promising therapeutic approach with a unique mechanism of action, as it only boosts eCB signaling where it is physiologically engaged rather than broadly stimulating CB1R, as THC-based therapies do.

## Connecting eCB impairment to synaptic and learning deficits

**Figure 1:**
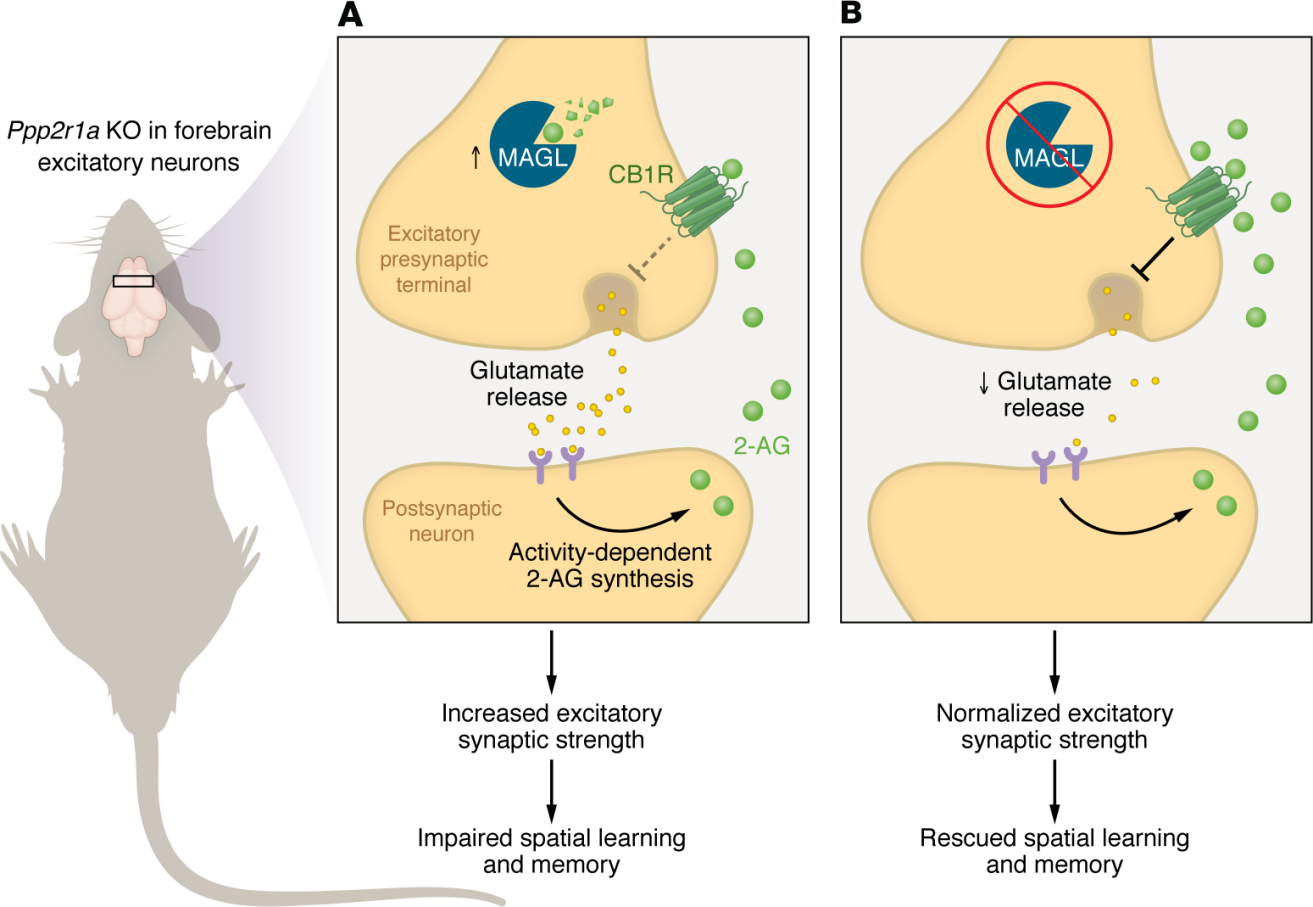
Impaired eCB signaling underlies synaptic alterations and spatial learning and memory deficits in *Ppp2r1a*-deficient mice. The eCB 2-AG is produced by postsynaptic neurons and normally acts on presynaptic CB1R to reduce glutamate release. Wang et al. leveraged the recently developed GRAB_eCB2.0_ sensor to detect the subcellular dynamics of 2-AG signaling via the fluorescence induced when extrasynaptic 2-AG binds to the sensor. (**A**) Heterozygotic genetic deletion of *Ppp2r1a* in mouse brain led to impaired 2-AG/CB1R signaling alongside increased excitatory synaptic strength and ensuing impairments in spatial learning in this model. (**B**) The sorted RNA-Seq data identified the upregulation of mRNA encoding for the presynaptic enzyme MAGL, which regulates tonic levels of 2-AG, in the *Ppp2r1a*-deficient mouse forebrain. Wang et al. showed that pharmacological inhibition of MAGL rescued 2-AG signaling, leading to normalization of excitatory synaptic strength and spatial learning and memory.

Children carrying genetic variants in the scaffolding subunit of the PP2A enzyme *PPP2R1A* exhibit symptoms typical of neurodevelopmental disorders, such as moderate-to-severe intellectual disability and developmental delay; but how *PPP2R1A* variants disrupt brain function has remained unclear. In this issue, Wang et al. show that the heterozygous deletion of the murine analog *Ppp2r1a* in forebrain excitatory neurons of mice (hereafter referred to as NEX-het-cKO mice) results in impairments in spatial learning and memory that model *PPP2R1A*-associated intellectual disability in humans (Figure 1A) (11). In NEX-het-cKO mice, heterozygous *Ppp2r1a* deletion increased excitatory synaptic strength. When RNA-Seq of the mouse forebrain tissue revealed a link between *Ppp2r1a* haploinsufficiency and upregulation of *Magl*, Wang and colleagues hypothesized that the eCB signaling system, and specifically 2-AG signaling, was involved in this change. Indeed, using fiber photometry measurements of a genetically encoded fluorescent eCB sensor, they observed disruption of 2-AG signaling in the NEX-het-cKO mouse forebrain. The increased excitatory synapse strength observed in NEX-het-cKO mice was driven by its increased presynaptic release probability, likely due to reduced levels of 2-AG that act at presynaptic CB1R, suggesting that normalizing 2-AG levels may ameliorate synaptic deficits in these mice. Accordingly, the MAGL inhibitor JZL184 restored both the synaptic and learning deficits in these mice (Figure 1B).

Although multiple studies have reported stimulus- and disease-associated changes in 2-AG levels in mouse brain, existing approaches were limited in cellular and temporal resolution, providing only an aggregate snapshot of its complex signaling dynamics. Here, Wang et al. have leveraged an innovative technology enabling measurement of rapid and transient changes in cell-specific production of eCBs: the GRAB_eCB_ sensors developed by the Li laboratory at Peking University (12). The recent development of fluorescence sensors based on GPCRs that are activated by lipid mediators has revolutionized our ability to study the subcellular dynamics of these hydrophobic signaling molecules (12). Such genetically encoded fluorescent sensors allow for real-time detection of changes in the levels of select endogenous signaling molecules (13). The GRAB_eCB2.0_ sensor introduces a circularly permuted green fluorescent protein into the third intracellular loop of CB1R to elicit fluorescence upon 2-AG binding (14). Thus, GRAB_eCB2.0_ detects changes in 2-AG levels with subsecond resolution in cells in culture, mouse brain slices, and in mouse brain during behavior (15–19). Using this approach was essential for Wang et al.’s discovery that the MAGL inhibitor, JZL184, increases 2-AG levels.

## Limitations and next steps

While inhibiting MAGL activity represents a promising therapeutic approach, one must consider limitations associated with chronic MAGL inhibition, which include behavioral side effects and tolerance linked to CB1R desensitization (20–23). A possible explanation is that MAGL controls tonic 2-AG levels and ensuing elevated activity at CB1R leads to desensitization (7). The enzyme ABHD6, which controls only the stimulated production of 2-AG, could be considered as an alternative target, and the efficacy of its inhibitors does not appear to undergo tolerance (7). As the sorted RNA-Seq results presented in this study were suggestive of an increase in FAAH function (11), which constitutes another mechanism for controlling eCB signaling, it is also worth considering whether targeting FAAH would also rescue cognitive impairment in the *Ppp2r1a*-haploinsufficient mouse model. Furthermore, Wang et al.’s sorted RNA-Seq results identified many additional mRNAs that were greatly increased by *Ppp2r1a* haploinsufficiency, and analysis of these mRNAs by clustering GeneSets might reveal additional signaling pathways disrupted in this model, highlighting additional potential therapeutic targets. An exciting new line of research will be to identify the molecular mechanism that links heterozygote deletion of PPP2R1A to changes in the expression of MAGL that control 2-AG signaling.

The extent to which PPP2R1A regulates eCB signaling in this mouse model is quite pronounced, providing a solid foundation for future biological and mechanistic studies as well as follow-up preclinical investigations. These endeavors will foster the development of potentially transformative therapeutic approaches for the treatment of neurodevelopmental disorders as they will act via unexplored mechanisms of action. Although the first steps down a new line of clinical investigation are always uncertain, this study represents a strong lead for developing eCB-based therapies with the potential to have tremendous effect on patients.

## Version 1 09/02/2025

Electronic publication
